# Surveillance for Western Equine Encephalitis, St. Louis Encephalitis, and West Nile Viruses Using Reverse Transcription Loop-Mediated Isothermal Amplification

**DOI:** 10.1371/journal.pone.0147962

**Published:** 2016-01-25

**Authors:** Sarah S. Wheeler, Cameron S. Ball, Stanley A. Langevin, Ying Fang, Lark L. Coffey, Robert J. Meagher

**Affiliations:** 1 University of California Davis, School of Veterinary Medicine, Department of Pathology, Microbiology and Immunology, Davis, California, United States of America; 2 Sandia National Laboratories, Biotechnology and Bioengineering Department, Livermore, California, United States of America; University of Texas Medical Branch, UNITED STATES

## Abstract

Collection of mosquitoes and testing for vector-borne viruses is a key surveillance activity that directly influences the vector control efforts of public health agencies, including determining when and where to apply insecticides. Vector control districts in California routinely monitor for three human pathogenic viruses including West Nile virus (WNV), Western equine encephalitis virus (WEEV), and St. Louis encephalitis virus (SLEV). Reverse transcription quantitative polymerase chain reaction (RT-qPCR) offers highly sensitive and specific detection of these three viruses in a single multiplex reaction, but this technique requires costly, specialized equipment that is generally only available in centralized public health laboratories. We report the use of reverse transcription loop-mediated isothermal amplification (RT-LAMP) to detect WNV, WEEV, and SLEV RNA extracted from pooled mosquito samples collected in California, including novel primer sets for specific detection of WEEV and SLEV, targeting the nonstructural protein 4 (nsP4) gene of WEEV and the 3’ untranslated region (3’-UTR) of SLEV. Our WEEV and SLEV RT-LAMP primers allowed detection of <0.1 PFU/reaction of their respective targets in <30 minutes, and exhibited high specificity without cross reactivity when tested against a panel of alphaviruses and flaviviruses. Furthermore, the SLEV primers do not cross-react with WNV, despite both viruses being closely related members of the Japanese encephalitis virus complex. The SLEV and WEEV primers can also be combined in a single RT-LAMP reaction, with discrimination between amplicons by melt curve analysis. Although RT-qPCR is approximately one order of magnitude more sensitive than RT-LAMP for all three targets, the RT-LAMP technique is less instrumentally intensive than RT-qPCR and provides a more cost-effective method of vector-borne virus surveillance.

## Introduction

Testing mosquito vectors for human pathogenic viruses like West Nile virus is a central feature of successful surveillance approaches and an early predictor of human epidemics. Most surveillance in the United States and increasingly in other regions uses molecular detection of viral RNAs, usually by reverse transcription polymerase chain reaction (RT-PCR) or quantitative real-time RT-PCR (RT-qPCR). The presence of vector-borne viruses in mosquitoes, together with other measures of activity including mosquito distribution and abundance, human cases, dead bird surveillance, meteorological parameters and (for some viruses) sentinel chicken seroconversions, are used to make vector control decisions including when and where to apply insecticides. Three medically important vector-borne viruses, West Nile virus (WNV), Western equine encephalitis virus (WEEV), and St. Louis encephalitis virus (SLEV) are endemic in California and cause sporadic seasonal outbreaks, primarily during the summer months.

WEEV incidence has declined in the last few decades [1] and has not been detected in California since 2003. SLEV was detected in field-caught mosquitoes in California in 2015, for the first time since the arrival of WNV in 2003 [2, 3]. SLEV also still occurs in Mexico and South America [4]. Despite the much higher prevalence of WNV in California since 2003, SLEV and WEEV are still included in routine mosquito surveillance since vector-borne viruses can exist in cryptic and sporadic transmission cycles without the detection of human or equine disease, as exemplified by the re-emergence of SLEV in 2015.

Vector-borne disease surveillance programs primarily rely on techniques such as virus isolation, standard RT-PCR, and RT-qPCR to detect the presence of vector-borne viruses in targeted mosquito vectors. While these methodologies are the gold standard for arbovirus disease surveillance, they require well-equipped laboratories and well-trained technicians, both of which are severely lacking in many low resource settings with high vector-borne disease burden. RT-qPCR is the most sensitive assay for detecting vector-borne viruses, but the technique generally requires expensive equipment, and high quality RNA purified from the mosquito samples. Loop-mediated isothermal amplification (LAMP) is an isothermal nucleic acid amplification technique that is a useful alternative to PCR for pathogen detection and diagnostics [5–8]. When coupled with reverse transcription, the LAMP methodology can also be utilized for detecting RNA targets [9]. LAMP requires 4 or 6 primers that recognize 6 or 8 binding sites, providing a highly specific assay. A key advantage to the LAMP reaction is simplicity: the entire reaction (including reverse transcription) proceeds at a single temperature, eliminating the need for a thermal cycler. Reactions can be monitored through a variety of outputs including generation of turbidity [10], fluorescence indicators [11, 12], or color change [13]. The technique is also robust for crude specimens, allowing successful amplification from minimally processed samples such as heat-treated blood [12, 14–16, 17]. As such, LAMP can often be performed without DNA or RNA extraction. These advantages lessen the dependence upon highly skilled labor and well-equipped laboratories, which are prerequisites for RT-qPCR.

In this report, we describe use of RT-LAMP to detect WNV, WEEV, and SLEV, and apply the technique to RNA extracted from *Culex* mosquitoes collected in California. RT-LAMP was previously demonstrated for detection of WNV by Parida *et al* [18], and detection kits for this virus based on RT-LAMP are commercially available from several vendors. RT-LAMP primer sets have also been reported for numerous other important mosquito-borne viruses such as Dengue virus (DENV) [19, 20], Japanese encephalitis virus (JEV) [21–23], chikungunya virus (CHIKV) [24], Rift Valley fever virus (RVFV) [25, 26], and yellow fever virus (YFV) [27]. Because RT-LAMP detection of SLEV and WEEV was not previously reported, RT-LAMP primer sets for these viruses were developed and tested for sensitivity and specificity. At the time of method development neither WEEV nor SLEV had been recently detected in California mosquito pools, and thus archived samples were used for RT-LAMP evaluation. WNV-positive mosquito pools collected in 2014 were used to verify the lack of cross-reactivity with new SLEV and WEEV primers. These WNV positive pools were also used to demonstrate the applicability of RT-LAMP for parallel detection of WNV, WEEV and SLEV, analogous to the RT-LAMP “detection array” previously reported for parallel detection of DENV serotypes 1–4, JEV, and WNV [28]. In addition, we demonstrate duplex detection of WEEV and SLEV in a single tube using melt curve analysis.

## Materials and Methods

### RT-LAMP primer design

**Fig 1:**
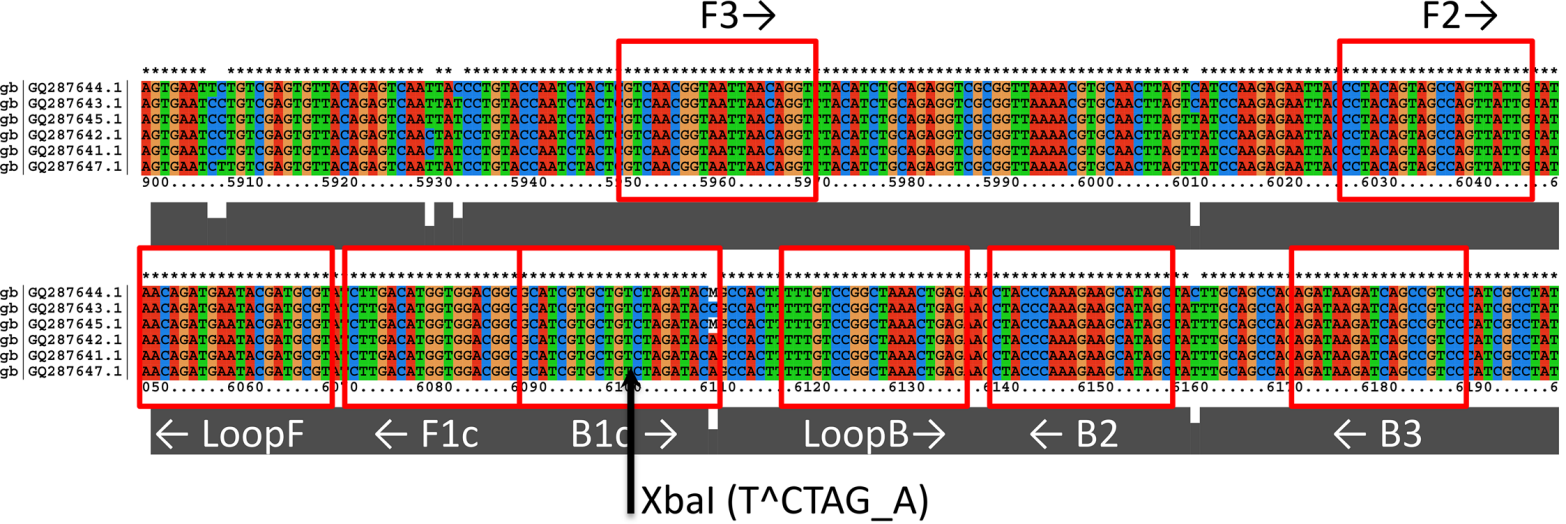
Sequence alignment showing priming regions for the WEEV nsP4 primer set. The arrow indicates the location of the *Xba*I restriction site. Genbank accession numbers refer to the following strains: GQ287644.1 = BFS2005; GQ287643.1 = Montana-64; GQ287645.1 = 71V1658; GQ287642.1 = Kern; GQ287641.1 = Imperial; GQ287647.1 = 85–452NM.

**Fig 2:**
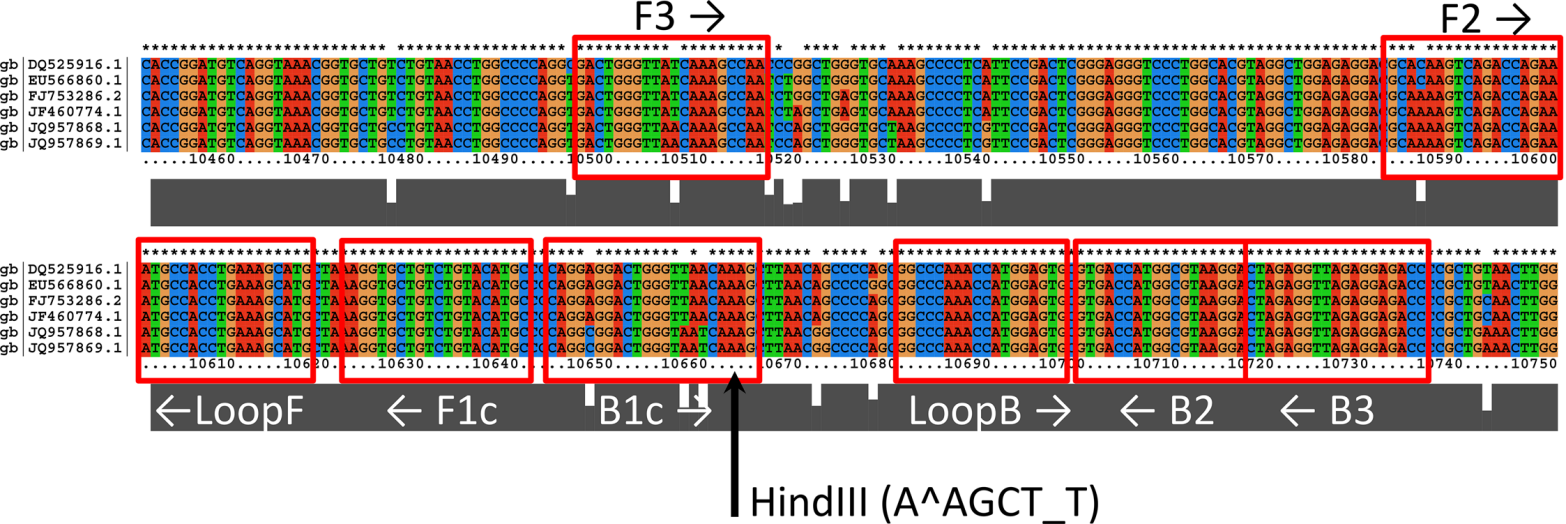
Sequence alignment showing priming regions for the SLEV 3’ UTR primer set. The arrow indicates the location of the *Hin*dIII restriction site.Genbank accession numbers refer to the following strains: DQ525916.1 = Kern 217; EU566860.1 = Hubbard; FJ753286.2 = CbaAr-4005; JF460774.1 = Imperial Valley; JQ957868.1 = Palenque-C475; JQ957869.1 = Palenque-A770.

Whole-genome sequence alignments for WEEV and SLEV were performed using ClustalX2. For WEEV, six North American strains were selected for which full-length or nearly full-length sequences were available in Genbank as of April, 2014 (a large number of additional WEEV genomes were deposited in Genbank after primers were designed [1]). For SLEV, 6 strains were selected for which full-length or nearly full-length sequences were available, representing primarily North American strains from Clade II in the tree developed by Kramer and Chandler [29]. Accession numbers of WEEV and SLEV strains used for primer design are presented in Figs 1 and 2.

Conserved regions of the viral genomes from the sequence alignments were identified. LAMP Designer v1.13 software (Premier Biosoft) was used to scan conserved regions for suitable LAMP primer sets using a single representative strain. Primer set candidates were then screened against the full set of aligned sequences to test for perfect or nearly perfect matches. Primer sets meeting this criterion were then analyzed by BLAST, first comparing to all WEEV or SLEV sequences (taxid 11039 and 11080, respectively) and then comparing to all viral sequences in Genbank, to determine likelihood of cross-reactivity with other viruses. Primer sets were also evaluated for hairpin formation, self-dimerization, and cross-dimerization.

### Viral culture

Selected viral strains used for sensitivity and specificity testing are listed in Table 1. These strains were pulled from a library of isolates archived at -80°C. From each isolate RNA was extracted and plaque assays were performed to obtain viral titers. RNA was extracted using a MagMax™ magnetic particle processor (Life Technologies, Grand Island, NY, USA), MagMAX™-96 Viral RNA Isolation Kit (Life Technologies, Grand Island, NY, USA), and manufacturer provided protocols. Plaque assays were performed using Vero cell cultures grown to confluence in six well plates, cultured with Dulbecco’s Modified Eagle Medium (DMEM) supplemented with 10% fetal bovine serum (FBS) and 500 U/mL penicillin and 0.5 mg/mL streptomycin, and maintained at 37°C and 5% CO_2_. Virus isolates were diluted in a 10-fold serial dilution, media was removed from each well of the 6-well plate and 200 uL of diluted virus was allowed to incubate on the monolayer for 60 min at 37°C. A double overlay system was used where the first overlay contained nutritive media and 5% agarose. The second overlay was applied and additionally contained 0.005% neutral red, the optimal time to count plaques occurred 1–3 days after the second overlay.

**Table 1:** Viral RNA used for testing sensitivity and specificity of RT-LAMP primers.

		RT-LAMP primer set (100 PFU target)		
Virus	Strain or Isolate Designation	SLEV 3’-UTR	WNV E gene	WEEV nsP4
*Flaviviruses*				
St. Louis encephalitis virus (SLEV)^1^	Kern217	+	–	–
	Bfs1750 (Ct 17.7)	+	–	–
	Ruls (Ct 12.9)	+	–	–
	Coav750 (Ct 23.9)	+	–	–
	69M1143 (Ct 13.7)	+	–	–
	BeAn246407 (Ct 20.4)	+	–	–
	CorAn9124 (PCR neg.)	+	–	–
	CorAn9275 (PCR neg.)	+	–^2^	–
West Nile virus (WNV)	L-CA-04 SAC-04-7168	–	+	–
Yellow fever virus (YFV)	17D	–	–	–
Rocio virus (ROCV)	SP H 34675	–	–	–
Usutu virus (USV)	SA AR 1776	–	–	–
Ilheus virus (ILHV)	Ilheus B44532	–	–	–
Dengue virus serotype 1 (DENV-1)	BC-796	–	–	–
Dengue virus serotype 2 (DENV-2)	BC-122-94	–	–	–
Dengue virus serotype 3 (DENV-3)	BC 156–97	–	–	–
*Alphaviruses*				
Western equine encephalitis virus (WEEV)^1^	KERN 5547	–	–	+
	Lake43 (Ct 14.9)	–	–	+
Sindbis virus (SINV)	EDS-14	–	–	–
Ross River virus (RRV)	SW 38457	–	–	–
Chikungunya virus (CHIKV)	Ross	–	–	–
Venezuelan equine encephalitis virus (VEEV)	TC-83	–^2^	–	–
Barmah Forest virus (BFV)	Barmah Forest (TVP-4119)	–	–	–
Highlands J virus (HJV)	WC-431	–	–	–
Eastern equine encephalitis virus (EEEV)^3^	EEEV/X/USA/A15072/2003	–	–	–

^1^ Isolatesof SLEV other than Kern 217, and WEEV isolate Lake43 were not quantitated by plaque assay. Extracted RNA was quantitated by RT-qPCR and used undiluted as a template in RT-LAMP. RT-qPCR Ct values are indicated. CorAn 9124 and CorAn9275 were not detected by RT-qPCR primers.

^2^ A single reaction showed positive amplification within 45 minutes of incubation (1 of 11 replicates for SLEV CorAn 9275 with WNV primers; 1 of 18 replicates for VEEV TC-83 with SLEV primers). Melt curve analysis indicates that these are isolated occurrences of cross-contamination with positive-control RNA.

^3^ Cross-reactivity testing for EEEV was performed with 10^6^ copies of an *in vitro* transcribed fragment of the EEEV nsP4 gene corresponding to the WEEV RT-LAMP target site including an additional 46 nt upstream and 65 nt downstream of the outer primer (F3 and B3) binding sites. The *in vitro* transcribed RNA represents nucleotides 5980–6329 (350 bases) of Genbank # KJ469613.1.

Due to select agent restrictions for work with Eastern equine encephalitis virus (EEEV), a segment of EEEV nsP4 RNA was purchased. The RNA was *in vitro* transcribed from a synthetic gene corresponding to nucleotides 5980–6329 (350 bases) of Genbank accession number KJ469613.1 (Biosynthesis, Lewisville, TX, USA). The transcript was purified to remove traces of DNA, and verified DNA-free by PCR (performed by the supplier).

### Mosquito pools

Mosquito pools were collected as part of routine mosquito surveillance performed by California vector control agencies. RNA from selected pools was obtained through the UC Davis Center for Vectorborne Disease Surveillance Labortory (CVEC) with the permission of the collecting agency. Pools comprised up to 50 conspecific, female, field-caught mosquitoes. RNA was extracted by MagMax^TM^ as described above.

### RT-qPCR

Detection of viral RNA in mosquito pools was performed at CVEC using a multiplex RT-qPCR for WNV, SLEV, and WEEV. This assay was performed using the primers and probes listed in S1 Table, except the probe for SLEV had a Quasar 670 reporter and Black Hole 2 Quencher (BHQ-2) and the probe for WEEV had a TAMRA reporter and BHQ-2. Reactions were completed on a ViiA 7 Real-Time PCR system (Life Technologies, Grand Island, NY, USA) using SensiFAST Probe Lo-ROX One-step Kits (Bioline, Taunton, MA, USA) and manufacturer recommended protocols. Additional RT-qPCR using the WNV, SLEV, and WEEV primer and probe sets (S1 Table) were performed on the RNA from selected virus isolates (Table 1) as part of the sensitivity and specificity testing at Sandia. These reactions were performed using a one-step RT-PCR kit (iTaq Universal Probes One-Step Kit, BioRad, Hercules, CA) and BioRad CFX96 instrument, with detection in the FAM channel. Total reaction volume was 10 μL and contained 500nM of each forward and reverse primer and 200nM of the probe. PCR was performed with an initial reverse transcription step at 50°C for 10 minutes, followed by an initial denaturation for 50 seconds, then 40 cycles of 95°C for 15 seconds and 60°C for 30 seconds followed by a plate read.

### RT-LAMP

RT-LAMP was performed in 10 μL reaction volumes in thin-walled PCR strip tubes or 96-well plates. The reaction mixture had a final composition (after adding water or template) of 1X Isothermal Amplification Buffer (New England Biolabs, NEB #B0537S) supplemented with an additional 6 mM MgSO4 (NEB #B1003S, final 8 mM MgSO_4_), 0.8 M Betaine (Sigma #B-0300), 1.4 mM each dNTP (NEB #N0447L), 0.32 units/μL Bst 2.0 WarmStart DNA polymerase (NEB #M0538M), 0.2 units/μL AMV reverse transcriptase (NEB #M0277T, or Life Science Advanced Technologies #AMVRTT-5), and 2 μM (or in some instances 4 μM) SYTO 82 detection dye (Life Technologies #S11363) [11]. Primers were used in the amounts typically recommended for LAMP: 0.2 μM each for outer primers F3 and B3; 1.6 μM each for inner primers FIP and BIP; and 0.8 μM each for loop primers LF and LB. RT-LAMP with real-time fluorescence monitoring was carried out in a BioRad CFX96, using detection channel 2 (HEX) for monitoring SYTO 82 dye. Reactions were incubated at a constant temperature of 63°C for 50–70 minutes, with plate read steps at intervals of 1 minute (in the BioRad CFX96, this is accomplished with a 48-second single-temperature cycle followed by a plate read which takes approximately 12 seconds in all-channel mode). Incubation was typically followed by inactivation of the enzyme at 95°C for 2 minutes, followed by cooling to 65°C for 2 minutes, and a melt curve from 65–95°C in 0.2°C increments. Time-to-positivity values were determined using the BioRad CFX Manager software, using baseline-subtracted curves, and a single threshold value auto-calculated by the CFX manager.

To demonstrate testing with simpler instrumentation, we also performed reactions using a portable isothermal fluorimeter (Optigene Genie III). The protocol was identical to that described above, except that SYTO 9 (Life Technologies #S34854) was used in place of SYTO 82, to ensure compatibility with the Genie III detection optics. A fluorescence photograph of the reaction endpoint (with SYTO 9) was taken using a blue LED flashlight (CREE WF-502B) and an amber-colored plastic filter (LEE Filters #158). Background fluorescence with SYTO 9 was minimized by photographing the tubes upon a hot plate set to 63°C.

Gel electrophoretic analysis of RT-LAMP products was performed using an Invitrogen e-Gel (2% agarose with ethidium bromide); 1 μL of RT-LAMP product was diluted with DI water to a total volume of 20 μL that was loaded onto the e-Gel for nucleic acid separation. In addition, to confirm specificity of amplification, 3 μL aliquots of products from positive control reactions were digested with 20 units of *Xba*I (for WEEV nsP4 RT-LAMP), or *Hind*III (for SLEV 3’-UTR RT-LAMP) for 1 hour at 37°C in 20 μL total reaction volume, using buffer supplied by the manufacturer (New England Biolabs). Digested products were also run on an e-Gel as described above.

### Sensitivity and Specificity Testing

SLEV, WEEV and WNV LAMP primers were tested for sensitivity and cross-reactivity against multiple conspecific strains and closely related viruses (Table 1). Sensitivity testing was performed with a dilution series of a single reference isolate of each virus: WEEV Kern 5547 (Genbank KJ554975.1), SLEV Kern 217 (Genbank DQ525916.1), and WNV L-CA-04 SAC-04-7168 (Genbank DQ080059.1). Conspecific strains for SLEV were chosen amongst the lineages described by Kramer et al. [27]. We tested isolates falling within Clades IA (Bfs1750), IB (Coav750), IIA(69M143), VA (BeAn246407), and VII (CorAn9124 and CorAn9275). One conspecific strain of WEEV (Lake 43, Genbank KJ554985) was also tested. All other WEEV isolates within the collection at UC Davis were identical to the Kern 5547 reference isolate within the primer binding regions. A panel of flaviviruses and alphaviruses (Table 1) was developed to test for cross-reactivity. Each primer set (SLEV, WEEV, and WNV) was tested against the entire panel, using 100 PFU equivalents of viral RNA template per reaction. Because of the select agent status of EEEV, primers were tested against an in vitro transcribed portion of nsP4 gene instead of genomic RNA (methods described above), with 10^6^ copies of RNA template per reaction.

### Multiplexed RT-LAMP with melt curve analysis

WNV, SLEV 3’-UTR, and WEEV-nsP4 primer sets were tested with mixtures of WNV L-CA-04 SAC-04-7168, SLEV Kern 217, and WEEV Kern 5547 RNA templates. Individual primer sets were combined in equal amounts to create four multiplexed reaction types: WNV + SLEV + WEEV, WNV + SLEV, WNV + WEEV, and SLEV + WEEV. Corresponding viral RNA templates were combined in equal parts and added to matching reaction mixes. Positive control reactions contained a total of 100 PFU equivalents of viral RNA template per reaction. Multiplexed reactions were performed in triplicate with the RT-LAMP protocol described above.

## Results and Discussion

### WEEV and SLEV primer design

Our primer design protocol identified two candidate primer sets for WEEV (targeting nsP1 and nsP4), and two candidate primer sets for SLEV (targeting the 3’-UTR and capsid C protein). According to the sequence alignment the 3’-UTR is well conserved among sequenced isolates of SLEV, and yet sufficiently distinct from other flaviviruses to allow specific detection of SLEV by RT-LAMP. The flavivirus 3’-UTR was previously targeted by LAMP primer sets designed for dengue serotypes 1–4 [19, 20]. Similarly, the nsP4 (RNA polymerase) gene of WEEV was among the most highly conserved region of that genome. The region targeted here shared 81% sequence identity with North American isolates of Eastern equine encephalitis virus (EEEV), but was <80% identical to any other alphaviruses.

In initial screening the WEEV-nsP4 and SLEV 3’-UTR sets provided faster amplification and lower detection limits than the WEEV-nsP1 and SLEV-C sets; therefore, only the nsP4 and 3’-UTR sets were evaluated further. The WEEV nsP4 and SLEV 3’-UTR primer sequences are shown in Table 2, and sequence alignments showing the positioning of the primers are presented in Figs 1 and 2. The less efficient WEEV nsP1 and SLEV C primer sequences are provided in S2 Table

**Table 2:** RT-LAMP Primers used herein.

Primer Name	Genome position^1^	Sequence^2^	Source
SLEV 3’ F3	10497–10516	GACTGGGTTAWCAAAGCCAA	New
SLEV 3’ B3	10736–10718	GGTCTCCTCTAACCTCTAG	
SLEV 3’ FIP(F1c + F2)	10641–10622;10583–10600	GCATGTACAGACAGCACCTT +GCACAAGTCAGACCAGAA	
SLEV 3’ BIP^3^(B1c + B2)	10644–10665;10717–10700	CAGGAGGACTGGGTTAACAAAG +TCCTTACGCCATGGTCAC	
SLEV 3’ LF	10618–10601	CATGCTTTCAGGTGGCAT	
SLEV 3’ LB	10681–10688	GGCCCAAACCATGGAGTG	
WEEV nsP4 F3	5949–5958	GTCAACGGTAATTAACAGGT	New
WEEV nsP4 B3	6187–6170	GGACGGCTGATCTTATCT	
WEEV nsP4 FIP(F1c + F2)	6087–6070;6025–6044	GCCGTCCACCATGTCAAG +CCTACAGTAGCCAGTTATTG	
WEEV nsP4 BIP(B1c + B2)	6088–6108;6156–6138	GCATCGTGCTGTCTAGATACM +GCTATGCTTCTTTGGGTAG	
WEEV nsP4 LF	6067–6048	ACGCATCGTATTCATCTGTT	
WEEV nsP4 LB	6116–6134	TTTGTCCGGCTAAACTGAG	
WNV F3	1028–1046	TGGATTTGGTTCTCGAAGG	Parida [18]
WNV B3	1228–1210	GGTCAGCACGTTTGTCATT	
WNV FIP(F1c + TTTT + F2)	1121–1100;1050–1069	TTGGCCGCCTCCATATTCATCA + TTTT +CAGCTGCGTGACTATCATGT	
WNV BIP(B1c + TTTT + B2)	1144–1165;1208–1190	TGCTATTTGGCTACCGTCAGCG + TTTT +TGAGCTTCTCCCATGGTCG	
WNV LF	1093–1075	CATCGATGGTAGGCTTGTC	
WNV LB	1169–1186	TCTCCACCAAAGCTGCGT	

^1^ Genome positions are based on SLEV strain Kern217 (Genbank DQ525916.1), WEEV strain Montana-64 (Genbank GQ287643.1), and (as per reference [18]) WNV strain NY99 (Genbank AF196835).

^2^ Sequence for inner primers FIP and BIP is shown separating out the F1c or B1c region (underlined) followed by the F2 or B2 region (not underlined). The complete FIP primer sequence is obtained by concatenating the F1c and F2 sequences (FIP = F1c + F2), and likewise for BIP. Primers designed by Parida *et al* [18] also include a TTTT linker between the two segments of the inner primers.

^3^ A second BIP primer with 3 single-base substitutions in the B1c region intended to cover additional SLEV strains was designed but not used for testing; the alternative sequence (BIP = B1c + B2) is CAGG**C**GGACTGGG**T**A**A**TCAAAG + TCCTTACGCCATGGTCAC. The alternative BIP sequence is a match for two Mexican isolates designated Palenque-C475 and Palenque-A770 (Genbank JQ957868.1 and JQ957869.1), which form a distinct phylogenetic clade [30].

**Fig 3:**
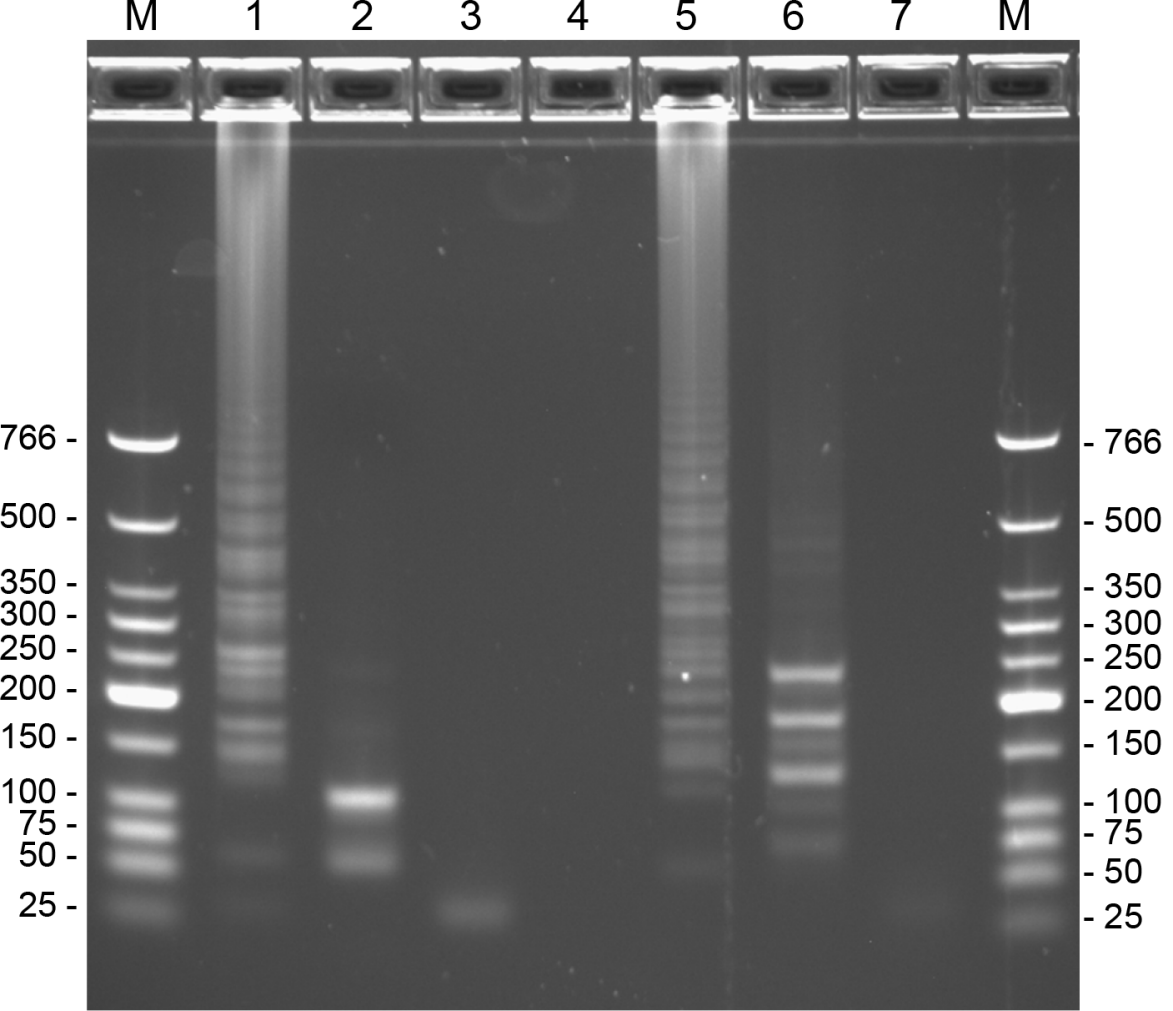
Electrophoretic analysis of RT-LAMP amplification products with WEEV nsP4 and SLEV 3’ UTR primer sets. The gel is a 2% agarose E-gel (Invitrogen) with ethidium bromide. The marker (“M”) is a Low Molecular Weight DNA Ladder (New England Biolabs). Lane 1: WEEV positive control reaction with 2 PFU equivalent WEEV RNA in 20 μL. Lane 2: WEEV positive control after digestion with *Xba*I. Lane 3: No-template control reaction with WEEV nsP4 primer set. Lane 4: no sample. Lane 5: SLEV positive control reaction with 2 PFU equivalent SLEV RNA in 20 μL. Lane 6: SLEV positive control after digestion with *Hin*dIII. Lane 7: No-template control reaction with SLEV 3’-UTR primer set. Cutting sites within the amplicon regions are shown in Figs 1 and 2.

Gel electrophoresis of the SLEV (3’-UTR) and WEEV (nsP4) RT-LAMP reaction products resulted in a prototypical ladder-like banding pattern typical of LAMP reactions (Fig 3, lanes 1 and 5). An online tool for LAMP restriction digest fragment analysis (http://creisle.github.io/creisle.lamprflp/) was used to predict restriction digest banding patterns based on the LAMP amplicon structure proposed by Notomi *et al* [6]. A restriction digest of the WEEV nsP4 amplicon with *XbaI* was predicted to yield main bands at 67 and 115 bp, with fainter bands at 50, 63, 77, and 97 bp. This is consistent with the banding pattern in Lane 2, which has primary bands slightly larger than 50 and 100 bp. Digestion of the SLEV 3’-UTR amplicon with *Hin*dIII was predicted to yield bright bands at 130, 185, and 240 bp, with fainter bands at 58, 62, 76, 80, 136, and 146 bp. This corresponds closely to the pattern of banding in Lane 6. Additional faint bands in these digests at higher molecular weight may be due to incomplete digestion, or the presence of other amplicon structures not proposed by Notomi *et al* [5, 6].

A recent survey of 33 WEEV strains [1] collected primarily in the United States indicated that all sampled strains were ~95% identical. The six strains used here for the sequence alignment were representative of three (Groups B1, B2, and B3) out of four of the WEEV lineages defined by Bergren et al. [1]. The nsP4 regions of these isolates had a single polymorphic site falling within a LAMP primer binding region. This mismatch occurred at the 3’ end of the B1c priming region. Based upon LAMP primer design guidelines provided by Eiken Genomics (http://loopamp.eiken.co.jp/e/lamp/primer.html), we infer that mismatches at the 3’ end of the B2, and the 5’ end of the B1c are more detrimental than mismatches at the 3’ end of B1c. However, since the effect of mismatches on LAMP performance is not well characterized, the polymorphic site was accommodated with a degenerate base (M) which covers all isolates used in the sequence alignment. After primer development, we did identify a single isolate of WEEV within the collection at UC Davis (Lake43, Genbank #KJ554985) with a single mismatch to the primer set, at the 5’ end of the F1c priming region. RNA from this isolate was successfully amplified, despite the mismatch (which also occurs in one other closely related WEEV isolate within Genbank, #KJ554984). The fourth WEEV lineage (Group A) described by Bergren et al. [1] comprised early WEEV isolates from 1930 and 1941. When we compared our primers to the “California” isolate (Genbank KJ554965.1, from the “A” lineage defined by Bergen et al. [1]), two mismatches were identified, one each near the middle of the F1c and B1c priming regions. The tolerance of LAMP to mismatches in the middle of priming regions is not well described. However, due to the high conservation of the target site within the WEEV nsP4 gene, our LAMP primers would likely detect most naturally occurring WEEV isolates.SLEV has a greater geographic distribution than WEEV, and sequences in Genbank show significantly higher divergence than is observed for sequenced isolates of WEEV. A phylogenetic tree based on the SLEV E gene sequences identified 7 clades [29]. Clades I and II represent primarily North American isolates, and clades III-VI represent primarily Central and South American isolates. The E gene is more divergent than the 3’ UTR region targeted by our primers. However, a comprehensive tree was not available for the 3’ UTR region, due to limited published sequences for SLEV that include the 3’ UTR. Sequence alignment for primer design included only North American isolates, and sensitivity testing was performed primarily with a single strain (Kern 217, Clade IIC). To determine whether the primer set was effective for detecting diverse lineages of SLEV, additional isolates falling within Clades IA (Bfs1750), IB (Coav750), IIA(69M143), VA (BeAn246407), and VII (CorAn9124 and CorAn9275) were tested. SLEV LAMP primers successfully detected RNA from all tested isolates, originating from both North and South America. In contrast the RT-qPCR assay used for surveillance in California failed to detect the two Clade VII isolates from Argentina, as indicated in Table 1

### Sensitivity and specificity of RT-LAMP for WEEV, SLEV, and WNV

**Fig 4:**
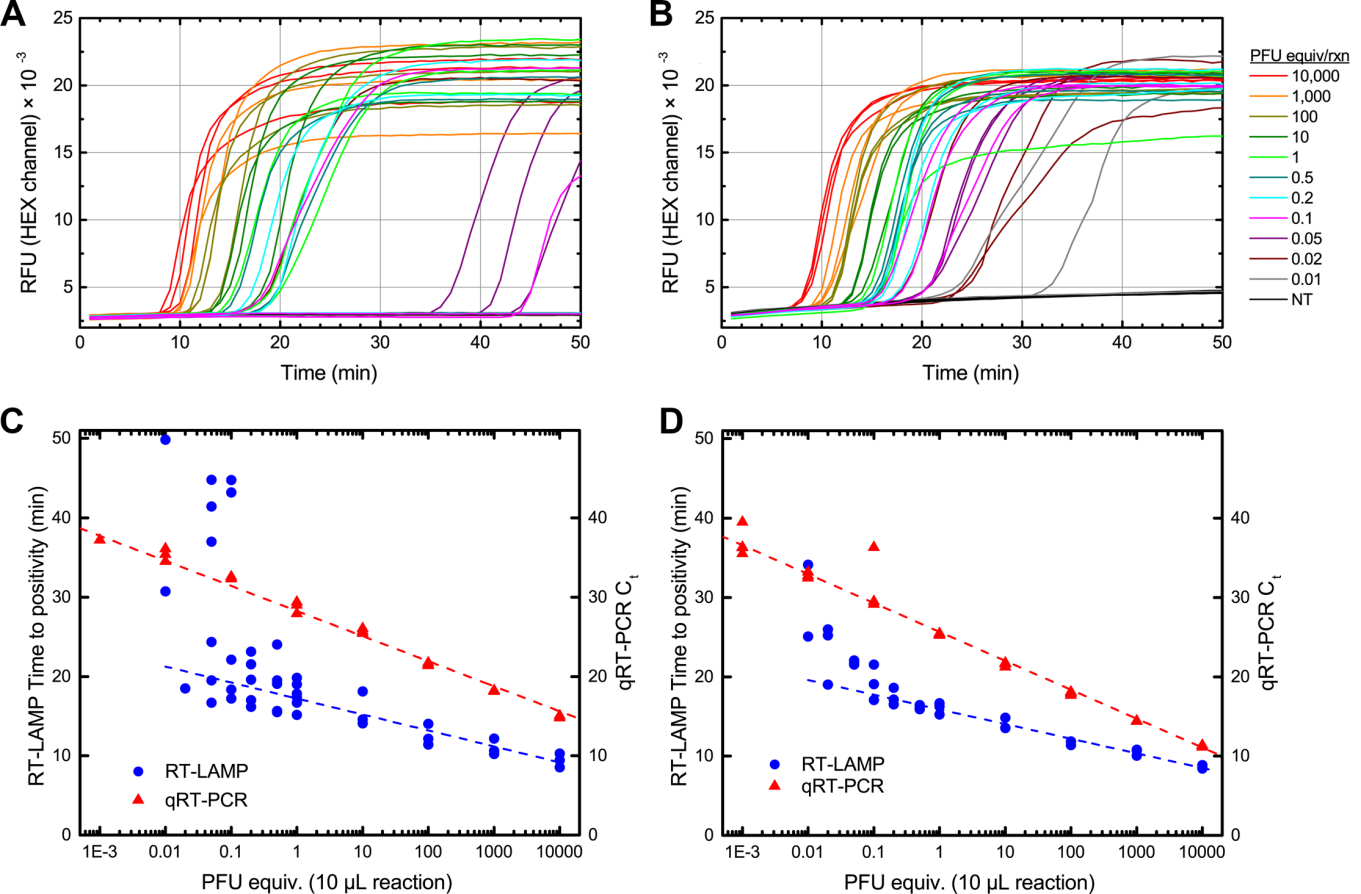
Quantitative analysis of SLEV and WEEV RT-LAMP. Panels A,B: Real-time RT-LAMP amplification curves for SLEV isolate Kern 217 (A) and WEEV isolate Kern 5547 (B). Reactions were performed in triplicate with viral RNA ranging from 10,000 PFU equivalents to 0.01 PFU equivalent per 10 μL reaction. Reactions were monitored in real-time using a BioRad CFX96 thermal cycler, using the HEX channel to monitor the signal from SYTO 82; the abbreviation “RFU” refers to Relative Fluorescence Units. Panels C,D: Standard curves for SLEV (C) and WEEV (D) generated by real-time monitoring of RT-LAMP (blue circles), versus RT-qPCR (red triangles). Time-to-positivity in RT-LAMP was determined by the BioRad CFX manager software, using baseline-subtracted quantitation data.

Real-time monitoring of RT-LAMP reactions for WEEV and SLEV primer sets was performed using serial dilutions of viral RNA, extracted from cultured virus. Fig 4 shows real-time monitoring curves for RT-LAMP reactions for each virus, as well as standard curves generated for these viral RNA by both RT-LAMP and RT-qPCR. In addition, a standard curve was generated using the published WNV RT-LAMP primer set [18] and was used for comparison to RT-qPCR; the resulting standard curves for WNV are shown in S1 Fig. All three RT-LAMP primer sets reliably detect their corresponding targets at 0.1 PFU equivalent, with less reliable detection down to 0.01 PFU. For all three viruses, RT-qPCR produced linear calibration curves to Ct >35, corresponding to viral titers of ~0.01 PFU, and less reliable detection down to 0.001 PFU. Overall, RT-LAMP reactions were 10-fold less sensitive than the corresponding RT-qPCR assays. The threshold of reliable detection for RT-LAMP was 100 PFU/mL corresponding to Ct ~30–32 by RT-qPCR. Positive detection generally occurred in 20–30 minutes, and often in <10 minutes for samples containing >10^4^ PFU/mL. Although RT-LAMP sensitivity was slightly lower than RT-qPCR, WNV RT-LAMP reactions were markedly more sensitive than commercially available antigen tests. Two common tests used for WNV surveillance are VecTest® WNV antigen assay (Medical Analysis Systems, Inc., Camarillo, CA) and Rapid Analyte Measurement Platform (RAMP®) WNV test (Response Biomedical, Corp., Burnaby British Columbia, Canada); these tests have minimum sensitivities of 10^5^ and 10^3^ PFU/mL, respectively.

Although real-time monitoring of LAMP by means such as turbidity or fluorescent dyes is common [10, 11], we note that real-time monitoring of RT-LAMP does not produce as precise a standard curve as RT-qPCR, particularly at lower viral RNA levels where time to positivity can vary substantially. We attribute this to the continuous nature of RT-LAMP, as opposed to the discrete cycling nature of RT-qPCR. In a well-designed RT-qPCR assay, each cycle in the exponential amplification regime leads to a reproducible doubling of the amount of amplicon. In RT-LAMP, however, slight variations in the early stages of the reaction can result in larger fluctuations in amplification time, particularly in the stochastic regime with few template copies, where multiple mechanisms may reduce the efficiency of the RT step in particular [31]. Real-time monitoring of RT-LAMP reactions can provide order-of-magnitude estimates of viral RNA concentration, but for precise quantitation RT-qPCR is preferable, particularly for low concentrations of viral RNA. The value of RT-LAMP for viral detection lies in the simplicity of the technique, particularly when applied in low-resource settings, where a yes-or-no answer determined by an endpoint measurement is sufficient.

Although the purpose of the current study was to develop and characterize new primer sets for SLEV and WEEV, we also demonstrated RT-LAMP detection of these targets using simpler instrumentation, as shown in S2 Fig. First, we carried out the RT-LAMP reactions for SLEV and WEEV using the Genie III, a portable isothermal fluorimeter with rechargeable battery and built-in wireless communication. Secondly, we took a photograph of the fluorescence endpoint using a handheld LED flashlight for illumination, and placing an amber-colored plastic film in front of the camera lens. In both cases, the fluorescence endpoint is clearly distinguishable between no-template control reactions and positive control reactions (tested down to 0.1 PFU equivalent of template).

For specificity testing, WEEV, SLEV, and WNV primer sets were tested against a panel of alphaviruses and flaviviruses, as indicated in Table 1. For WEEV, the closest relatives are other New World alphaviruses, Venezuelan equine encephalitis virus (VEEV) and Eastern equine encephalitis virus (EEEV). We found no cross-reactivity between any of the primer sets among non-target viruses. The only unexpected positive reactions were two isolated instances that we attribute to cross-contamination, where RNA from positive controls was inadvertently introduced into non-target cross-reactivity reactions. The specific instances (noted in Table 1) are a single positive reaction (out of 18 replicates) when VEEV TC-83 RNA template was tested with the SLEV primers, and a single positive reaction (out of 11 replicates) when SLEV strain CorAn 9275 was tested with WNV primers. In both cases, the single positive amplifications displayed identical melt curves to positive-control reactions performed on the same plate, and these isolated positive results could not be reproduced. Otherwise no evidence of cross-reactivity between primers sets and non-target viruses was observed. In addition, there was no evidence of non-specific amplification in no-template control reactions with the SLEV and WEEV primer sets incubated for up to 50 minutes. Previously, non-specific amplification was observed with the previously published WNV primer set, but most often developed after > 50 minutes of incubation.

We tested archived RNA from 12 pooled mosquito samples collected in California in 2002 or 2014 that were positive by RT-qPCR for WEEV, SLEV, or WNV, or negative for all three, to determine if our RT-LAMP assays could detect these viruses in mosquito samples. Results of these tests are presented in Table 3. The RT-LAMP assay detected SLEV in four pools that had RT-qPCR Cts ranging from 24.4 to 27.7. The RT-LAMP assay also detected WEEV in 5 pools that had RT-qPCR Cts ranging from 22.8 to 26.5, but failed to detect WEEV in one sample with a Ct value of 32, which is below the sensitivity demonstrated for the WEEV RT-LAMP assays. Sample sizes were limited, but the new primer sets readily detected SLEV and WEEV in mosquito pool RNA. Although sensitivity was slightly reduced compared to RT-qPCR, the specificity of these new primers was absolute.

**Table 3:** Analysis of mosquito pools by SLEV, WEEV, and WNV RT-LAMP.

	RT-qPCR Ct^1^			RT-LAMP Time-to-positivity (min)^2^		
Sample^3^	SLEV	WEEV	WNV	SLEV	WEEV	WNV
2002 COAV 490	–	22.8	–	–	12.2, 12.3	–
2002 COAV 512	–	26.5	–	–	14.0, 15.9	–
2002 COAV 513	–	25.1	–	–	13.0, 14.6	–
2002 COAV 984	–	23.0	–	–	12.1, 12.3	–
2002 COAV 942	–	24.5	–	–	12.2, 12.3	–
2002 COAV 862	27.7	–	–	13.1, 13.9	–	–
2002 IMPR 136	24.4	–	–	12.4, 12.5	–	–
2002 IMPR 139	26.5	–	–	13.4, 14.0	–	–
2002 IMPR 145	27.3	32.0	–	19.1, 21.3	–	–
2014 KERN 14–163	–	–	23.0	–	–	10.8, 11.1
2014 MARN 14–300	–	–	25.0	–	–	13.3, 13.8
2014 SUYA 14–144	–	–	–	–	–	–

^1^Triplex RT-qPCRfor SLEV, WEEV, and WNV surveillance samples performed by UC Davis Center for Vectorborne Diseases.

^2^Results of two replicates reported

^3^Sample designations indicate year and location of sample collection within California: COAV = Coachella Valley, IMPR = Imperial Valley; KERN = Kern County; MARN = Marin-Sonoma counties, SUYA = Sutter-Yuba counties.

### Multiplexing RT-LAMP by melt curve analysis

**Fig 5:**
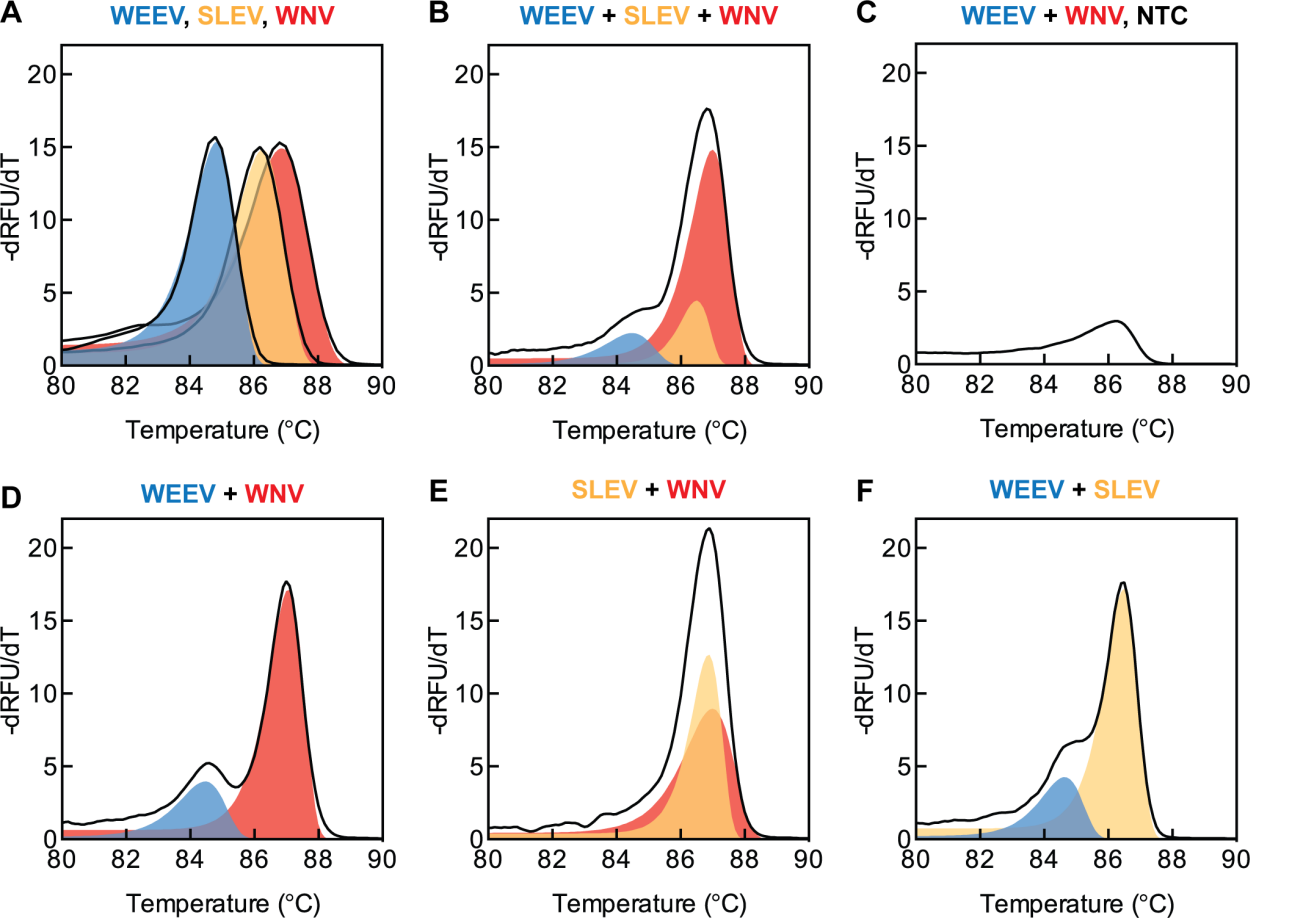
Melt derivative curves for singleplex and multiplex RT-LAMP. Solid black lines show average data from triplicate runs. Colored shaded areas represent the contributions of individual RT-LAMP targets to the overall signals to aid visualization. Blue = WEEV. Yellow = SLEV. Red = WNV. A) Singleplex reactions. B) Three-way combination. Only two peaks are distinguishable by eye. C) WEEV + WNV no template control. A relatively small melting peak is present, corresponding to spurious amplification after 40 min. D-F) Pairwise combinations. A smaller WEEV peak is distinguishable from WNV and SLEV peaks in D and F. WNV and SLEV peaks are indistinguishable by eye in E.

Multiplexed amplification by LAMP was consistent, and all positive control RT-LAMP reactions amplified target RNA in less than 30 minutes. No-template controls remained negative for 40 minutes, after which non-specific amplification was detected in multiplexes containing WNV and WEEV primer sets together. Amplification in WNV + SLEV + WEEV and WNV + WEEV no-template controls likely resulted from primer-dimer formation between the WNV BIP and the WEEV FIP primers. Negative control reactions containing the combination of WNV and WEEV primer sets showed left-skewed melting peaks at 86.3°C, corresponding to melting of amplified primer-dimers. The intensity of the negative control melt curves was lower in reactions with a lower initial concentration of WNV/WEEV primer sets. Positive controls were distinguishable from negative controls and produced LAMP products that were suitable for melt curve analysis. Examples are presented in Fig 5.

The melting curves of multiplexed LAMP reactions contained up to two distinguishable peaks for target identification. The first peak (84.5–84.9°C) corresponded to the melting of WEEV-specific amplicons. The second peak (86.5–87°C) corresponds to the melting of WNV-specific amplicons, SLEV-specific amplicons, or their combination.

The center and width of amplicon melting distributions determined the ease of multiplexed target detection. For example, the melting point of WNV amplicons was 0.5°C higher than that of SLEV amplicons. Because this difference is close to the 0.2°C accuracy limit of the real-time PCR instrument, reactions containing both WNV and SLEV amplicons displayed a single melting curve—with an intermediate melting temperature—instead of two discrete curves. Thus, simple peak picking was insufficient to differentiate WNV from SLEV in reactions containing both WNV and SLEV primer sets.

Furthermore, the relative intensity of discrete melt peaks reflected the relative abundance of individual LAMP products. However, in reactions with equal starting amounts of template, the WEEV amplicon melt-curve intensity was less than that of either the WNV or SLEV amplicons. This suggests that amplification with the WEEV primer set is slower or less efficient than either the WNV or SLEV primer sets. We have not tested experimentally whether this lower efficiency of amplification for WEEV reduces the detection limit of WEEV when present together in the same sample with a larger amount of WNV or SLEV.

Given the tendency to non-specific amplification observed with the combination of the WNV and WEEV primers, and the overlap of the WNV and SLEV melt curves, we conclude that only the WEEV and SLEV assays could be multiplexed in a single reaction. A more complex curve fitting algorithm based on parameters such as peak width, skew and temperature may be capable of robust differentiation of WEEV, SLEV, and WNV amplicons in multiplexed LAMP reactions. However, to our knowledge such an approach has not been demonstrated for LAMP melt curve analysis.

## Conclusion

The newly developed RT-LAMP primer sets for SLEV and WEEV show sensitivity and specificity comparable to a previously reported primer set for WNV [16]. Although the RT-LAMP assays are approximately an order of magnitude less sensitive than the corresponding RT-qPCR assays, the assay consistently detected to 0.1 PFU/reaction equivalents without cross-reactivity between related viruses. In addition, the RT-LAMP method can be greatly simplified compared to standard RT-qPCR reactions. Here purified RNA targets and a conventional real-time PCR thermal cycler were used to characterize RT-LAMP reactions, but we also demonstrate compatibility with a low-cost portable fluorimeter for monitoring isothermal amplification reactions, as well as compatibility with non-instrumented detection Future work is directly at developing simplified RT-LAMP protocols for RNA viruses such as WNV, SLEV, and WEEV. New protocols will utilize the tolerance of the Bst DNA polymerase to potential “PCR inhibitors” that are often present in crude samples such as mosquito or tissue homogenates. The combination of simplified instrumentation, minimal sample prep, no RNA extraction, and low reagent cost may enable the development of RT-LAMP based assays that are as simple to use as commercially available antigen tests. This would enable highly sensitive and specific surveillance for vector-borne diseases in basic laboratories or field settings where RT-qPCR resources are unavailable.

## Supporting Information

S1 Fig Standard curves for WNV RT-LAMP and RT-qPCR. RT-LAMP time to positivity (blue circles) versus RT-qPCR Ct (red triangles), for WNV isolate L-CA-04 SAC-04-7168 using the RT-LAMP primers published by Parida et al. [1], and the RT-qPCR primers published by Lanciotti et al [2]. At least three technical replicates were performed for each template concentration.
(PDF)

S2 Fig Detection of SLEV and WEEV by RT-LAMP using simplified techniques. (A) SLEV and WEEV RNA were detected by RT-LAMP using the OptiGene Genie III, a portable, battery-operated real-time fluorimeter. The screenshot in the inset of panel (A) shows real-time amplification curves generated for eight reactions, which are specified in Panel B. Reactions 1–4 use the SLEV 3’ UTR primer set, with 10, 1, 0.1 PFU equivalents of SLEV RNA (Reaction 4 is an SLEV no-template control). Reactions 5–8 use the WEEV nsP4 primer set, with 10, 1, 0.1 PFU equivalents of WEEV RNA (Reaction 8 is a WEEV no-template control). Panel (B) illustrates the endpoint of the same eight reactions, after incubation at 63°C for 30 minutes. To take this photograph, the strip of tubes was set upon a dark background, on a hotplate set at 63°C. Fluorescence was excited using a handheld blue LED flashlight, and a sheet of amber plastic film (LEE Filters #158) was placed in front of the lens of a digital camera. No contrast adjustment was applied. In Panel A, a barcode label affixed to the instrument has been pixelated to obscure identification.
(TIF)

S1 Table RT-qPCR primers used for quantitation of Western equine encephalitis virus (WEEV), St. Louis encephalitis virus (SLEV), and West Nile virus (WNV) viral RNA. IBFQ = Iowa Black FQ; BHQ1 = Black Hole Quencher 1 (The different quenchers have similar performance when paired with FAM, and do not affect the results of the PCR assays). The primer set names from the original literature references are provided in the table next to the reference numbers.
(PDF)

S2 Table Additional RT-LAMP primer sets for SLEV and WEEV. (PDF)
