# Supplementary material for: Surveillance for Western Equine Encephalitis, St. Louis Encephalitis, and West Nile Viruses Using Reverse Transcription Loop-Mediated Isothermal Amplification
Source: PLoS One. 2016 Jan 25;11(1):e0147962. doi: 10.1371/journal.pone.0147962 (PMC4726549; doi:10.1371/journal.pone.0147962)
Supplement: S1 Fig — RT-LAMP time to positivity (blue circles) versus RT-qPCR Ct (red triangles), for WNV isolate L-CA-04 SAC-04-7168 using the RT-LAMP primers published by Parida et al. [1], and the RT-qPCR primers published by Lanciotti et al [2]. At least three technical replicates were performed for each template concentration. (PDF) [file pone.0147962.s001.pdf]

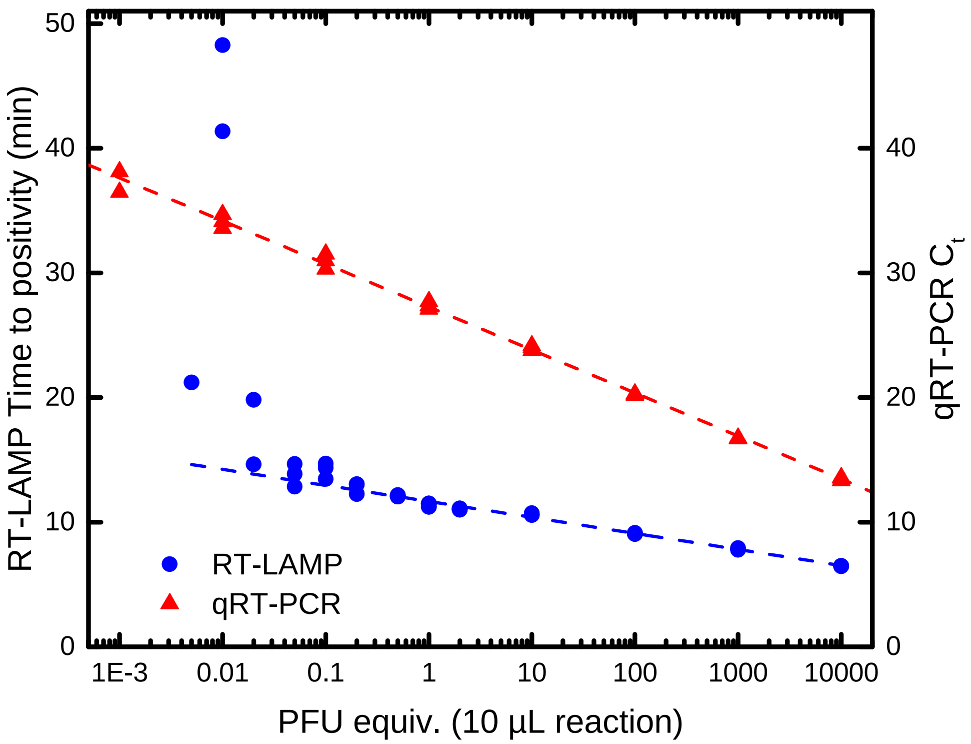

S1 Figure: RT-LAMP time to positivity (blue circles) versus RT-qPCR Ct (red triangles), for WNV isolate L-CA-04 SAC-04-7168 using the RT-LAMP primers published by Parida *et al.* [1], and the RT-qPCR primers published by Lanciotti *et al* [2]. At least three technical replicates were performed for each template concentration.

References for S1 Figure

1. Parida M, Posadas G, Inoue S, Hasebe F, Morita K (2004) Real-time reverse transcription loop-mediated isothermal amplification for rapid detection of West Nile virus. J Clin Microbiol 42: 257-263.

2. Lanciotti RS, Kerst AJ, Nasci RS, Godsey MS, Mitchell CJ, et al. (2000) Rapid detection of west nile virus from human clinical specimens, field-collected mosquitoes, and avian samples by a TaqMan reverse transcriptase-PCR assay. J Clin Microbiol 38: 4066-4071.
