# Supplementary figures and images for: Surveillance for Western Equine Encephalitis, St. Louis Encephalitis, and West Nile Viruses Using Reverse Transcription Loop-Mediated Isothermal Amplification
Source: PLoS One. 2016 Jan 25;11(1):e0147962. doi: 10.1371/journal.pone.0147962 (PMC4726549; doi:10.1371/journal.pone.0147962)

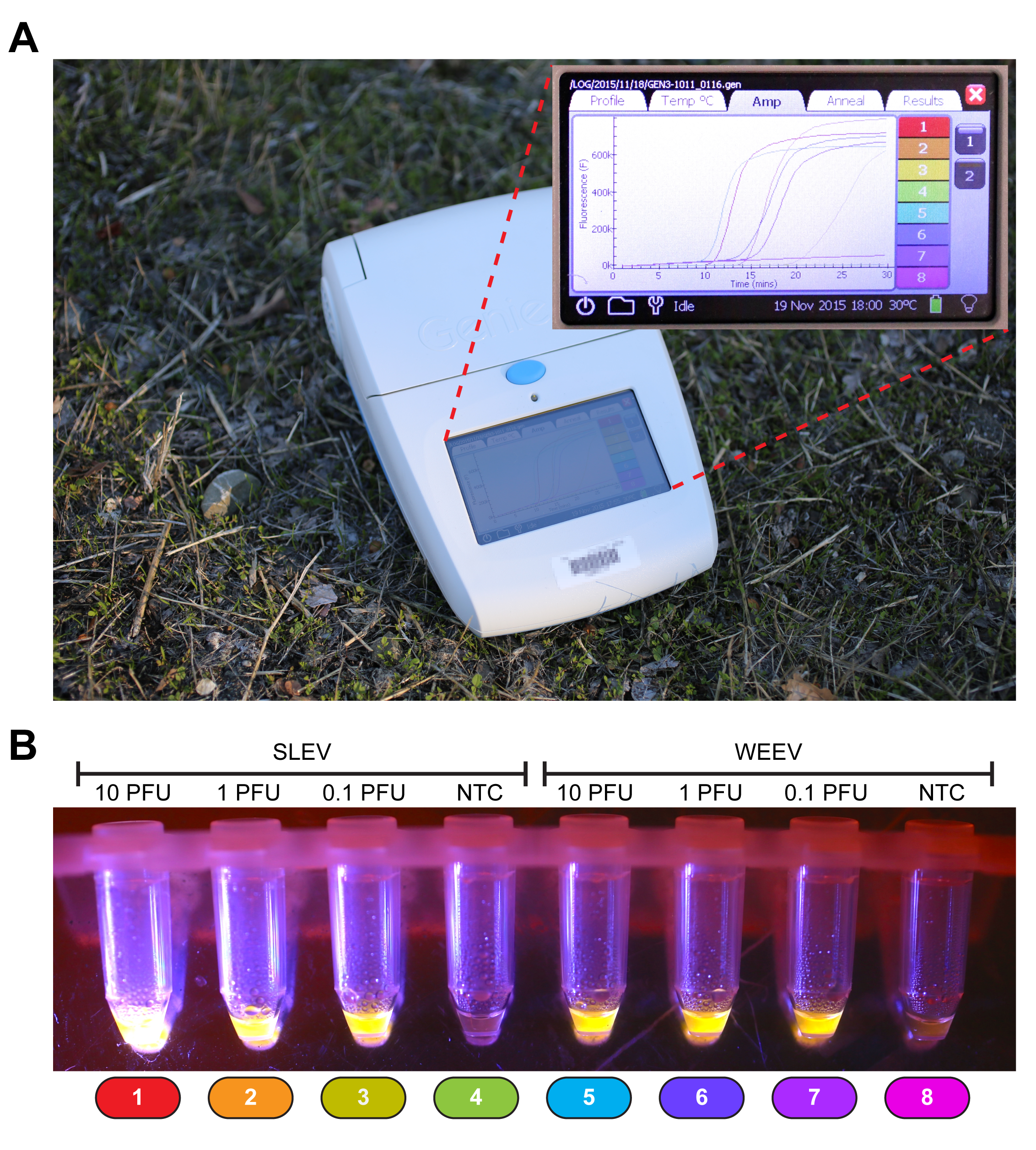

Supplement: S2 Fig — (A) SLEV and WEEV RNA were detected by RT-LAMP using the OptiGene Genie III, a portable, battery-operated real-time fluorimeter. The screenshot in the inset of panel (A) shows real-time amplification curves generated for eight reactions, which are specified in Panel B. Reactions 1–4 use the SLEV 3’ UTR primer set, with 10, 1, 0.1 PFU equivalents of SLEV RNA (Reaction 4 is an SLEV no-template control). Reactions 5–8 use the WEEV nsP4 primer set, with 10, 1, 0.1 PFU equivalents of WEEV RNA (Reaction 8 is a WEEV no-template control). Panel (B) illustrates the endpoint of the same eight reactions, after incubation at 63°C for 30 minutes. To take this photograph, the strip of tubes was set upon a dark background, on a hotplate set at 63°C. Fluorescence was excited using a handheld blue LED flashlight, and a sheet of amber plastic film (LEE Filters #158) was placed in front of the lens of a digital camera. No contrast adjustment was applied. In Panel A, a barcode label affixed to the instrument has been pixelated to obscure identification. (TIF) [file pone.0147962.s002.tif]
