# Supplementary material for: Surveillance for Western Equine Encephalitis, St. Louis Encephalitis, and West Nile Viruses Using Reverse Transcription Loop-Mediated Isothermal Amplification
Source: PLoS One. 2016 Jan 25;11(1):e0147962. doi: 10.1371/journal.pone.0147962 (PMC4726549; doi:10.1371/journal.pone.0147962)
Supplement: S1 Table — IBFQ = Iowa Black FQ; BHQ1 = Black Hole Quencher 1 (The different quenchers have similar performance when paired with FAM, and do not affect the results of the PCR assays). The primer set names from the original literature references are provided in the table next to the reference numbers. (PDF) [file pone.0147962.s003.pdf]

S1 Table: RT-qPCR primers used for quantitation of Western equine encephalitis virus (WEEV), St. Louis encephalitis virus (SLEV), and West Nile virus (WNV) viral RNA. IBFQ = Iowa Black FQ; BHQ1 = Black Hole Quencher 1 (The different quenchers have similar performance when paired with FAM, and do not affect the results of the PCR assays). The primer set names from the original literature references are provided in the table next to the reference numbers.

Primer/probe name	Sequence	Reference
WEEV-forward	AGG TAA ACT GCA CAT TCC ATT CC	[1] (WEEV-2)
WEEV-reverse	TTC GTG ACT GTA GGC GTG TGA	
WEEV-probe	FAM-CCG ACA GTC TGC CCG GTT CCG-IBFQ	
SLEV-forward	CTG GCT GTC GGA GGG ATT CT	[2] (SLE2420,
SLEV-reverse	TAG GTC AAT TGC ACA TCC CG	SLE2487c, and
SLEV-probe	FAM-TCT GGC GAC CAG CGT GCA AGC CG-IBFQ	SLE2444probe)
WNV-forward	TCA GCG ATC TCT CCA CCA AAG	[3] (WNENV)
WNV-reverse	GGG TCA GCA CGT TTG TCA TTG	
WNV-probe	FAM-TGC CCG ACC ATG GGA GAA GCTC -BHQ1	

References for S1 Table

1. Brault AC, Fang Y, Reisen WK (2015) Multiplex qRT-PCR for the Detection of Western Equine Encephalomyelitis, St. Louis Encephalitis, and West Nile Viral RNA in Mosquito Pools (Diptera: Culicidae). J Med Entomol 52: 491-499.

2. Lanciotti RS, Kerst AJ (2001) Nucleic Acid Sequence-Based Amplification Assays for Rapid Detection of West Nile and St. Louis Encephalitis Viruses. Journal of Clinical Microbiology 39: 4506-4513.

3. Lanciotti RS, Kerst AJ, Nasci RS, Godsey MS, Mitchell CJ, et al. (2000) Rapid detection of west nile virus from human clinical specimens, field-collected mosquitoes, and avian samples by a TaqMan reverse transcriptase-PCR assay. J Clin Microbiol 38: 4066-4071.
