# Supplementary material for: Surveillance for Western Equine Encephalitis, St. Louis Encephalitis, and West Nile Viruses Using Reverse Transcription Loop-Mediated Isothermal Amplification
Source: PLoS One. 2016 Jan 25;11(1):e0147962. doi: 10.1371/journal.pone.0147962 (PMC4726549; doi:10.1371/journal.pone.0147962)
Supplement: S2 Table — (PDF) [file pone.0147962.s004.pdf]

S2 Table: Additional RT-LAMP primer sets for SLEV and WEEV

Primer Name	Genome position[1]	Sequence[2]	Source
SLEV-C F3	176-193	GTAGAAACCGGGTTGTCAATA	New
SLEV-C B3	383-364	GATGGTGTCTAGCATRGATC	
SLEV-C FIP	247-227;	ATGGCTAGTATGAACCGCACG +	
(F1c + F2)	176-193	CTTGACGGGGTTGAAGAG	
SLEV-C BIP[3]	268-288;	CAGCTCTACAGCCAACTGAGG +	
(B1c + B2)	340-323	AGGTGTTTCAATGCCGTT	
SLEV-C LF	215-194	ATCCAGCAGACTTCCAAGTATC	
SLEV-C LB	290-307	GCTGAAGCGCAGATGGAG	
WEEV nsP1 F3	1376-1394	CCGTCTTTGACTCATTTGT	New
WEEV nsP1 B3	1643-1626	CCTGCCTCTTGCATAATG	
WEEV nsP1 FIP	1529-1512;	TCTTGCTGTAAGCCACGC +	
(F1c + F2)	1455-1472;	GCTTGAACCAACTGTCAA	
WEEV nsP1 BIP	1549-1568	GCGGAAGAGATCAGAGAAGC +	
(B1c + B2)	1621-1604	CTACTTCTGCCTCTACGG	
WEEV nsP1 LF	1493-1476	ATTGTAATAGCCGGTGCG	
WEEV nsP1 LB	1576-1594	CCCTTGCTCCCTGAAATAG	

[1] Genome positions are based on SLEV strain Kern217 (Genbank DQ525916.1) and WEEV strain Montana-64 (Genbank GQ287643.1).
